# Physical activity participation and the risk of chronic diseases among South Asian adults: protocol for a systematic review and meta-analysis

**DOI:** 10.1186/s13643-018-0848-9

**Published:** 2018-10-30

**Authors:** Susan Paudel, Alice J. Owen, Ebenezer Owusu-Addo, Ben J. Smith

**Affiliations:** 1 School of Public Health and Preventive Medicine, Monash University, 553 St Kilda Road, Melbourne, Victoria Australia; 2 Nepal Development Society, Kaski, Nepal; 3 Bureau of Integrated Rural Development, Kwame Nkrumah University of Science and Technology, Kumasi, Ghana; 4 Sydney School of Public Health, The University of Sydney, Sydney, Australia

**Keywords:** Physical activity, Chronic diseases, Systematic review, South Asia, Adults

## Abstract

**Background:**

Chronic diseases contribute to about half of the adult disease burden in the South Asian region. Meanwhile, physical activity levels are declining despite the global evidence of its role in the prevention of chronic diseases. While there are a plethora of systematic reviews on the effects of physical activity on chronic diseases, there has not yet been a synthesis of the evidence concerning the nature of this relationship among people living in South Asian countries incorporating multiple chronic diseases and a focus on physical activity domains. The aim of this protocol is to describe the rationale and methods for a systematic review of published research to identify the association between physical activity and selected chronic diseases and their markers and analysis of the strength of association with a focus on physical activity domains among South Asian adults 40 years and older.

**Methods:**

Nine electronic databases including Medline, PsycINFO, Embase, CENTRAL, CINAHL Plus, AgeLine, SPORTDiscus, Scopus and Web of Science will be systematically searched for papers reporting the association between physical activity and selected chronic diseases (type 2 diabetes mellitus, breast cancer, colorectal cancer, coronary heart disease, stroke, vascular diseases and musculoskeletal diseases (osteoarthritis, osteoporosis, back and neck pain)) and their markers using predefined search terms. Searches will be limited to peer-reviewed, English language papers with a quantitative design. In addition, a manual search of references of relevant systematic reviews as well as citations and references of eligible studies will also be carried out. The methodological appraisal will be performed using the National Institutes of Health quality assessment checklist for observational studies and the Effective Public Health Practice Project quality assessment tool for intervention studies. The overall quality of evidence for the study outcomes across the study designs will be assessed using the Grading of Recommendations Assessment, Development and Evaluation (GRADE) framework. The review results will be presented in the form of narrative synthesis, and a random effects meta-analysis is planned depending on the nature of included studies and available data.

**Discussion:**

This review will summarise the strength of the association between physical activity and selected chronic diseases and their markers among South Asian adults 40 years or older. The findings will provide an evidence base to guide public health policy and interventions in the South Asian region and to inform future research to address the rising burden of chronic diseases.

**Systematic review registration:**

PROSPERO CRD42018096505

**Electronic supplementary material:**

The online version of this article (10.1186/s13643-018-0848-9) contains supplementary material, which is available to authorized users.

## Background

South Asia (SA) is the home to nearly one fourth of the global population with nearly half of them living below the poverty line with limited access to health services [1]. The epidemiological transition is a common phenomenon across all countries in the region: Afghanistan, Bangladesh, Bhutan, India, Maldives, Nepal, Pakistan and Sri Lanka [1, 2]. In 2012, the World Health Organization (WHO) estimated that chronic diseases accounted for 37% of total deaths in Afghanistan, 50% in Pakistan, 56% in Bhutan, 59% in Bangladesh, 60% in India and Nepal, 75% in Sri Lanka and 81% in the Maldives [3]. The proportional mortality due to chronic diseases has increased in all these countries between 2008 and 2012, ranging from an increase of 3% in the Maldives to 28% in Afghanistan [3, 4]. Furthermore, the burden is expected to increase in the future [1], with population ageing, haphazard urbanisation and economic development driving sedentary behaviours and consumption of energy-dense foods [5].

Adults with low levels of physical activity (PA) are at an increased risk of chronic diseases and their risk markers [6, 7]. In addition to its independent effect, the link between PA and obesity further increases its public health significance [8]. The Global Burden of Disease study found that 9.9%, 2.5% and 1.1% of total coronary heart disease (CHD), diabetes and breast cancer-related disability-adjusted life years (DALYs) in SA is attributable to low PA [9]. However, available data show wide variations in PA levels both between and within countries of SA. A study carried out in four regions of India (Tamil Nadu, Maharashtra, Jharkhand and Chandigarh) among 14,000 individuals aged 20 years and above reported that one in two Indians (54%) are inactive [10, 11]. In Bhutan, the national survey of chronic diseases risk factors found that 6.4% of 40–69 years old adults did not meet the WHO recommendations of at least 150 min of moderate-intensity physical activity per week in 2014 [12]. In some countries, the prevalence of inactivity has been found to be higher among females and in urban areas [10, 13].

Work- and transport-related activities are the main contributors to total PA in South Asians [12–14]. The 2015 Sri Lankan STEPwise approach to noncommunicable disease surveillance (STEPS) survey reported the prevalence of work- and transport-related physical activity to be 67% and 57%, respectively, among 45–59 years adults [14]. Only 5% of Sri Lankan adults of the same age group engaged in some form of recreational physical activity [14].

Global evidence exists for the effect of leisure time PA in minimising the risk for chronic diseases or their markers [15–18]. The association is stronger for leisure time and activities of daily living than for occupational and transport-related activities [19]. However, available evidence shows that these forms of activities are less common in SA [12–14]. This emphasises the need for context-specific evidence on the role of regular forms of occupation-, household- and transport-related activities in chronic disease prevention in the region. There has not yet been a systematic review of the association between multiple chronic diseases and regular PA among South Asian adults. Previous reviews have either focused on South Asian migrants in western nations [20], have studied one or two health outcomes only [21–23], been limited to selected countries [23, 24] or included younger people only [25]. Considering the present trends, inactivity levels will likely increase in the future and this will have significant implications for the burden of chronic diseases in the region. Understanding the relationship between PA and chronic diseases among South Asian adults is crucial to informing public health policy and interventions to tackle the chronic disease burden in the region.

## Research objective and questions

The objective of this protocol is to systematically review published research to examine the association between PA and selected chronic diseases and their markers and to provide a summary estimate of the strength of association among South Asian adults 40 years and older. Specifically, the review aims to answer the following research questions:

1. What is the association between total PA and chronic diseases and their risk markers among South Asian adults 40 years and older?
2. What is the association between PA domains (transport, household, occupational and leisure) and the outcome variables of interest (chronic diseases and their risk markers)?

## Methods

The proposed review will be guided by the Preferred Reporting Items for Systematic Reviews and Meta-Analyses (PRISMA) statement to ensure transparency in the study selection and reporting process [26, 27]. The review protocol has been developed using the PRISMA Protocols 2015 (PRISMA-P-2015) [28, 29], which is available in Additional file 1. This protocol will guide the review, and deviations (if any) will be reported along with the reasons for the changes in the methods used. The review is registered with PROSPERO International Prospective Register of Ongoing Systematic Reviews (CRD42018096505). It is exempted from the ethical review process as it only uses published de-identified data and does not involve any direct contact with the human participants.

### Outcome measure

The primary outcome variable of interest is the occurrence of chronic disease. For this review, the following chronic diseases will be considered: type 2 diabetes mellitus (T2DM), breast cancer, colorectal cancer, cardiovascular disease (CHD, stroke, vascular diseases) and musculoskeletal diseases (osteoarthritis, osteoporosis, back and neck pain). Chronic disease markers (body weight, body mass index, blood sugar, blood pressure, lipids, cholesterol, bone mass density and the metabolic syndrome) are the secondary outcomes of interest.

### Exposure measure

PA is the primary exposure variable of interest which focuses on routine PA. It refers to work-, household-, leisure- or active commuting-related activities of varying intensities that are carried out during normal life. The review will not include activities that are carried out in a controlled, structured environment for research (e.g. supervised exercise on treadmills or yoga sessions). Sedentary behaviour including screen time is the secondary exposure variable of interest.

### Information sources and search strategy

To identify published, peer-reviewed, English language papers that have studied the association between PA and chronic diseases, a systematic search of nine electronic databases will be carried out, namely Medline, Embase, PsycINFO, CENTRAL, CINAHL Plus, SPORTDiscus, AgeLine, Scopus and the Web of Science. Search terms related to PA and sedentary behaviour, chronic diseases and their markers and South Asia will be used. In accordance with the PICO (Population, Intervention of interest, Control and Outcome) framework, the question guiding this review is “In South Asian adults 40 years and older, what is the effect of participation in routine PA on chronic diseases or their risk markers?” A Medical librarian experienced in database searching for systematic reviews will be consulted to develop the search strategy. A draft search strategy will be tested to ensure all previously identified papers are captured and then finalised with necessary modifications. Additional file 2 includes the initial search strategy used in some of these databases.

To ensure that all relevant studies are captured, a manual search of references of relevant systematic reviews as well as citations and references of eligible studies will also be carried out. Considering the relatively recent interest in PA research in the South Asian region, searches will be limited to papers published from 2000 onwards.

### Inclusion criteria

Quantitative studies among South Asian adults aged 40 years or older that have reported the association between PA or sedentary behaviour and chronic diseases or their markers will be included. Studies must be conducted in at least one of the following South Asian countries: Nepal, India, Pakistan, Sri Lanka, Bangladesh, Afghanistan, Maldives and India. Multi-country studies with no specific results for any of the South Asian countries and studies among South Asian migrants will be excluded. Adults 40 years and older will be included in this review considering the declining physical activity and increasing sedentary time with age [30] along with an early onset of chronic disease such as acute myocardial infarction among South Asian adults [31]. Studies involving adults with no sub-group-specific results for those 40 years and above will also be excluded.

Observational and population-level intervention studies investigating PA or sedentary behaviour in routine circumstances as exposure will be included. Population-level intervention studies, which do not control the research environment (such as mass media or health education campaigns), measuring routine PA and reporting associations specific to this type of activity will be included. Studies carried out in many settings such as community, workplace or hospitals will be included, provided they have studied PA in routine circumstances. Review articles, qualitative studies and non-peer-reviewed (grey) literature will be excluded.

### Study selection

All the identified papers will initially be exported to Endnote X8 software [32]. After removing duplicates, the non-duplicate records will then be exported to Covidence systematic review software (available at www.covidence.org) for further screening of titles, abstracts and full texts. Initially, the records will be screened for their titles which will involve exclusion of all irrelevant titles. This will then be followed by the review of abstracts against the inclusion/exclusion criteria by two reviewers. Full texts of the abstracts considered eligible will then be retrieved and reviewed by two reviewers. The final decision regarding inclusion of the studies will be made through joint discussion and consultation with the review team. Team members will not be blinded to author and journal details. Corresponding authors will be contacted if any further information is required to determine study eligibility. The PRISMA flowchart will be used to report the outcomes of screening [33].

### Data extraction and management

A Microsoft Excel data extraction template (Additional file 3) has been developed which will be used by one author to extract information regarding authors, publication date, country of origin, study population, sample size, outcome measures, exposure variables, types of PA, measures of association and the study findings from all the eligible studies. Extracted data will be verified by another reviewer. Any discrepancy will be resolved jointly within the review team. Two attempts of contacting corresponding authors will be made through emails to obtain missing or additional information. If no response is obtained in these two attempts, the review will use only the information reported in the papers.

### Quality appraisal

After determination of study eligibility, two reviewers will independently assess the quality of intervention studies using the Effective Public Health Practice Project (EPHPP) quality assessment tool [34]. Reviewers will rate papers on each of the six criteria (selection bias, allocation bias, confounder control, blinding, data collection methods and drop-outs) as weak, moderate or strong which will then be used to determine the final rating [34]. The National Institutes for Health (NIH) checklist will be used for observational studies [35]. The NIH has developed separate checklists for case-control studies and cohort/cross-sectional studies and uses 14 criteria to assess the quality: research question, study population, participation rate, inclusion criteria, sample size, exposure prior to outcome, sufficient time frame, different levels of exposure, exposure measures, multiple exposure assessment, outcome measures, blinding, follow-up rate and statistical analyses [35]. All studies will be given a “yes”, “no” or “not applicable” for each of these criteria. The responses to each criterion will be compared between the two reviewers, and any discrepancy resolved by discussion and consensus within the review team. Percentages for “yes” scores will be calculated, and studies will be categorised as poor, fair or good quality. Studies with lower than 50% “yes” score will be considered as poor, those with 50–75% will be considered as fair and those with greater than 75% will be considered good quality [36]. Criteria that are not applicable (such as loss to follow-up after baseline in case of cross-sectional studies) will not be considered for calculating the percentages.

The Grading of Recommendations, Assessment, Development and Evaluation (GRADE) framework will be used to grade the quality of evidence for each outcome as high, moderate, low or very low. Across the study designs, randomised trials will be rated as high and cross-sectional studies as low quality. The evidence will be downgraded if there is a severe risk of bias, inconsistency in the study results, indirectness or imprecision [37].

### Data analysis and reporting

A generic variance-based random effects meta-analysis will be carried out to determine the association between PA and chronic diseases and their markers. Though the studies might be comparable to allow for pooling the effect sizes, certain variations might exist in participants’ characteristics and study features which might result in different effect sizes across the studies. Hence, random effects meta-analysis will be used to obtain a summary estimate with the assumption that the true effects will be normally distributed [38]. It is anticipated that studies will report different measures of associations (such as correlation coefficients or mean difference) and use different PA categorisation. These effect estimates (Fisher’s *z* or Cohen’s *d*) will be converted to log odds ratio using the methods and formulae described by Borenstein et al. [38] to obtain a common metric, provided the studies are comparable in other characteristics. Odds ratio (OR) will be the measure of association, and an alpha level of 0.05 will be considered statistically significant. Adjusted associations will be used for pooling the effect size. However, reported or calculated unadjusted estimates will also be used if adjusted values are not available. The *I*^2^ statistic, which denotes the percentage of variation across the studies due to heterogeneity, will be calculated to examine heterogeneity of the study results [39]. *I*^2^ value of 25%, 50% and 75% will denote low, moderate and high heterogeneity respectively [39].

We anticipate that sometimes a single study might contribute more than one effect estimates. It might report results for multiple outcomes or for the single outcome at multiple time points or different groups of participants. If the sub-groups are independent of each other, they will be treated as a separate study and all the independent estimates will be used in the meta-analysis [38]. However, if the study reports estimate for multiple outcomes or time points using the same group of participants, only one estimate per study will be used. In case of multiple outcomes, only the estimates for primary outcome variable will be used while for multiple time points, the estimates for the latest follow-up point will be used. When there are multiple articles from a single study, the article with the most relevant data will be used. Sensitivity analysis will be performed on all outcome categories by removing one study at a time to ensure that the study findings are not influenced by a study. Sub-group analysis is planned across geographical sub-regions (such as India vs non-India) and study design to examine their potential influence on the reported outcomes. A funnel plot will be plotted to assess publication bias. All statistical analyses will be performed using Review Manager version 5 software (RevMan 5) [40].

If meta-analysis is not feasible, a narrative summary with supporting tables will be presented to describe the nature and pattern of associations. The strength of evidence for each association from all reviewed studies will be reported as positive, negative or non-significant. The outcome variables of interest will be grouped into three categories for reporting the review findings: cardio-metabolic conditions (T2DM, CHD, Hypertension (HTN), metabolic syndrome, obesity measures), musculoskeletal conditions (osteoporosis, neck and back pain) and cancer (breast and colorectal cancer). These three categories will also include the relevant risk markers. Corresponding authors will be contacted for missing data or if additional information is required to calculate the effect sizes. If these attempts are unsuccessful, only the narrative synthesis will be provided for the study.

## Discussion

Studies from high-income countries dominate the current evidence on the role of PA in the prevention of chronic diseases, and most of these report on the impact of leisure time and supervised PA. By contrast, the primary forms of PA in the South Asian region appear to be transport- and work-related physical activities. The low engagement in leisure-time physical activities in South Asian countries may in part be attributed to inadequate availability of well-maintained parks and playgrounds [41]. There is currently limited knowledge about the contribution of different types of physical activities to the prevention of major chronic diseases in South Asia, and the need for this is heightened by the demographic and epidemiological changes taking place in several countries in the region.

The Global Action Plan on Physical Activity (GAPPA) 2018 highlights the importance of walking and cycling as crucial contributors to regular PA while noting the widespread decline in these types of activity, particularly in low- and middle-income countries (LMICs), due to the increasing preference for motorised transport [42]. The report further states that different population sub-groups may have preferences for different forms of physical activity in line with their culture, context and resources [42]. Notwithstanding the benefits of modifying the built environment to enable different types of activity, identifying the current and accessible forms of physical activity that reduce chronic disease risk is valuable for policy and program development.

Recognising the global distribution of the chronic disease burden, the GAPPA report argues that stronger evidence is needed concerning PA prevalence and outcomes from LMICs [42]. To contribute to addressing the knowledge gap, this review will identify the association between selected chronic diseases and PA among South Asian adults 40 years or older. This information will be important to update the current understanding of the role of different PA domains in preventing chronic diseases among South Asian adults. Considering the role of PA in the prevention of chronic diseases [6, 7], the findings will provide an evidence base to plan public health interventions to encourage adults to adopt an active life and decrease their sedentary behaviours.

The inclusion of multiple chronic diseases and their risk markers and the focus on PA carried out in routine circumstances are major strengths of this review. Exclusion of grey literature might be a potential limitation as this could omit useful information arising from unpublished sources. However, the risks associated with the validity and reliability of the information arising from non-peer-reviewed sources were the basis for excluding these reports. Heterogeneity between the studies regarding PA domains, their categorisation and measurement methods, and study population differences may reduce the potential to derive conclusions concerning PA and chronic disease associations. We anticipate identifying research gaps and recommending priorities for future research.

## Additional files

Additional file 1: PRISMA Protocol 2015. (DOCX 31 kb)

Additional file 2: Initial search strategy. (DOCX 37 kb)

Additional file 3: Microsoft Excel data extraction template. (DOCX 13 kb)

## Abbreviations

CENTRAL: Cochrane Central Register of Controlled Trials
CHD: Coronary heart disease
DALYs: Disability-adjusted life years
EPHPP: Effective Public Health Practice Project
GAPPA: Global Action Plan on Physical Activity
GRADE: Grading of Recommendations Assessment, Development and Evaluation
NIH: National Institutes for Health
PA: Physical activity
PRISMA: Preferred Reporting Items for Systematic Reviews and Meta-analyses
PRISMA-P: Preferred Reporting Items for Systematic Review and Meta-analysis Protocols
PROSPERO: International Prospective Register of Systematic Reviews
SA: South Asia
STEPS: STEPwise approach to noncommunicable disease surveillance
T2DM: Type 2 diabetes mellitus
WHO: World Health Organization
