# Supplementary material for: Physical activity participation and the risk of chronic diseases among South Asian adults: protocol for a systematic review and meta-analysis
Source: Syst Rev. 2018 Oct 30;7:177. doi: 10.1186/s13643-018-0848-9 (PMC6208083; doi:10.1186/s13643-018-0848-9)
Supplement: Supplementary file 2 — Initial search strategy. (DOCX 37 kb) [file 13643_2018_848_MOESM2_ESM.docx]

Title of the database searched: Ovid MEDLINE(R) Epub Ahead of Print, In-Process & Other Non-Indexed Citations, Ovid MEDLINE(R) Daily, Ovid MEDLINE and Versions(R) 1946 to March 14, 2018 
Name of the software platform: Ovid

Date search was run: 18 March 2018

Limits used: English language, humans, age group: 19+, journal articles

Search Strategy:

| # | Searches | Results |
| --- | --- | --- |
| 1 | bangladesh/ or bhutan/ or india/ or nepal/ or pakistan/ or sri lanka/ | 123041 |
| 2 | (bangladesh* or bhutan* or india* or afghan* or nepal* or pakistan* or sri lanka* or srilanka* or maldiv* or south asia* or southasia*).mp. | 243160 |
| 3 | 1 or 2 | 243160 |
| 4 | exercise/ or muscle stretching exercises/ or resistance training/ or running/ or jogging/ or swimming/ or walking/ or stair climbing/ or leisure activities/ or recreation/ or dancing/ or gardening/ or games, recreational/ or video games/ or sports/ or physical fitness/ or bicycling/ | 209480 |
| 5 | (physical activit* or physical inactiv* or exercise* or nonexerciser* or non exerciser* or muscle stretching exercise* or resistance train* or run* or jog* or swim* or walk* or stair* climb* or leisure activit* or recreation or danc* or gardening or games, recreational or sport* or physical fitness* or cycle* or bicycl*).mp. | 1287470 |
| 6 | "Physical Education and Training"/ or Motor Activity/ or life style/ or sedentary lifestyle/ | 156874 |
| 7 | (physical education or fitness* or motor activity).mp. | 185211 |
| 8 | (lifestyle* or life style* or sitting time).mp. | 121344 |
| 9 | (sedentary adj1 (lifestyle* or activit* or behavio?r* or time)).mp. | 12706 |
| 10 | TELEVISION/ or Computers, Handheld/ or SMARTPHONE/ or Cell Phone/ | 24081 |
| 11 | (computer* or smartphone* or mobile phone* or cell phone* or television* or TV).mp. | 768086 |
| 12 | (leisure adj3 activit*).mp. | 12667 |
| 13 | ((household or domestic) adj activit*).mp. | 792 |
| 14 | (leisure adj2 exercise*).mp. | 449 |
| 15 | ((TV or television) adj3 (watch* or view*or time)).mp. | 2729 |
| 16 | ((screen or screen based) adj2 (time or view*)).mp. | 1857 |
| 17 | 4 or 5 or 6 or 7 or 8 or 9 or 10 or 11 or 12 or 13 or 14 or 15 or 16 | 2188959 |
| 18 | diabetes mellitus, type 2/ | 111829 |
| 19 | chronic disease/ or noncommunicable diseases/ | 246642 |
| 20 | (chronic disease* or noncommunicable disease* or non communicable disease*).mp. | 291459 |
| 21 | ((type II or type 2) adj diabetes).mp. | 108488 |
| 22 | (T2D or NIDDM or noninsulin or non insulin).mp. | 21904 |
| 23 | coronary disease/ or coronary artery disease/ or myocardial infarction/ | 313408 |
| 24 | stroke/ or brain infarction/ | 85134 |
| 25 | Breast Neoplasms/ or colorectal neoplasms/ or musculoskeletal diseases/ | 338017 |
| 26 | ((breast or colon or colorectal) adj3 (cancer* or tumo?r* or neoplasm*)).mp. | 476115 |
| 27 | (coronary disease* or coronary artery disease* or myocardial infarction or myocardial isch?emia or stroke* or cerebrovascular infarction or cerebrovascular accident or musculoskeletal disease* or brain infarction).mp. | 656045 |
| 28 | ((coronary or heart) adj2 disease*).mp. | 408235 |
| 29 | Vascular Diseases/ or Peripheral Vascular Diseases/ or Peripheral Arterial Disease/ | 48804 |
| 30 | (vascular disease* or peripheral vascular disease* or peripheral arter* disease*).mp. | 86761 |
| 31 | LOW BACK PAIN/ or BACK PAIN/ or NECK PAIN/ | 39444 |
| 32 | OSTEOARTHRITIS/ | 33363 |
| 33 | OSTEOPOROSIS, POSTMENOPAUSAL/ or OSTEOPOROSIS/ | 50905 |
| 34 | (low back pain or neck pain or back pain or osteoarthritis or osteoporosis).mp. | 209731 |
| 35 | 18 or 19 or 20 or 21 or 22 or 23 or 24 or 25 or 26 or 27 or 28 or 29 or 30 or 31 or 32 or 33 or 34 | 1951435 |
| 36 | body weight/ or overweight/ or obesity/ or adiposity/ or body mass index/ or waist-hip ratio/ | 388880 |
| 37 | (body weight or overweight or obes* or adipos* or body mass index or BMI or waist-hip ratio or waist circumference* or waist-height ratio).mp. | 778557 |
| 38 | ((abdominal or body) adj2 fat).mp. | 44115 |
| 39 | Blood Pressure/ or blood glucose/ or lipids/ or triglycerides/ or metabolic syndrome/ or waist circumference/ or waist-height ratio/ | 559868 |
| 40 | HYPERTENSION/ or hyperglycemia/ or glucose intolerance/ or hyperinsulinism/ or insulin resistance/ or CHOLESTEROL, LDL/ or CHOLESTEROL/ or CHOLESTEROL, HDL/ | 428054 |
| 41 | (blood adj (glucose or sugar* or pressure)).mp. | 575970 |
| 42 | (lipid* or triglycer* or hypertensi* or hyperglycemi* or glucose intoleran* or hyperinsulin* or insulin resistan* or cholesterol* or fatty acid* or fatty tissue* or glyc?emic index or hypercholesterol* or metabolic syndrome).mp. | 1384690 |
| 43 | (pressure adj (diastolic or systolic)).mp. | 2464 |
| 44 | 36 or 37 or 38 or 39 or 40 or 41 or 42 or 43 | 2227587 |
| 45 | 35 or 44 | 3807739 |
| 46 | 3 and 17 and 45 | 4706 |
| 47 | limit 46 to (english language and humans and yr="2000 -Current" and ("adult (19 to 44 years)" or "middle aged (45 plus years)") and journal article) | 1926 |

Note: # 3 indicates records related to South Asia, #17 indicates records related to physical activity, # 45 indicates records related to chronic disease or their markers, # 46 indicates records related to South Asia and physical activity and chronic disease/markers

Title of the database searched: Embase Classic+Embase 1947 to 2018 March 16

Name of the software platform: Ovid

Date search was run: 18 March 2018

Limits used: English language, human, age group: 18+, journal articles and articles in press, exclude Medline journals

Search Strategy:

| # | Searches | Results |
| --- | --- | --- |
| 1 | south asia/ or afghanistan/ or bangladesh/ or bhutan/ or india/ or nepal/ or pakistan/ or sri lanka/ | 181880 |
| 2 | (bangladesh* or bhutan* or india* or afghan* or nepal* or pakistan* or sri lanka* or srilanka* or maldiv* or south asia* or southasia*).mp. | 342561 |
| 3 | 1 or 2 | 342582 |
| 4 | sedentary lifestyle/ or lifestyle/ or physical activity/ or cycling/ or jogging/ or running/ or swimming/ or walking/ or sport/ or yoga/ or leisure/ or recreation/ or gardening/ or fitness/ or motor activity/ | 461827 |
| 5 | (lifestyle* or life style* or sitting time).mp. | 181265 |
| 6 | (sedentary adj1 (lifestyle* or activit* or behavio?r* or time)).mp. | 17607 |
| 7 | (computer* or smartphone* or mobile phone* or cell phone* or television* or TV).mp. | 1482194 |
| 8 | television viewing/ or television/ or smartphone/ or mobile phone/ | 36001 |
| 9 | (leisure adj3 activit*).mp. | 9822 |
| 10 | ((household or domestic) adj activit*).mp. | 1036 |
| 11 | (leisure adj2 exercise*).mp. | 611 |
| 12 | ((TV or television) adj3 (watch* or view*or time)).mp. | 3647 |
| 13 | ((screen or screen based) adj2 (time or view*)).mp. | 2428 |
| 14 | (physical activit* or physical inactiv* or exercise* or nonexerciser* or non exerciser* or muscle stretching exercise* or resistance train* or run* or jog* or swim* or walk* or stair* climb* or leisure activit* or recreation or danc* or gardening or game* or sport* or physical fitness* or cycle* or bicycl* or motor activit*).mp. | 1977284 |
| 15 | 4 or 5 or 6 or 7 or 8 or 9 or 10 or 11 or 12 or 13 or 14 | 3494920 |
| 16 | heart disease/ or ischemic heart disease/ or myocardial disease/ or chronic disease/ or non insulin dependent diabetes mellitus/ or non communicable disease/ | 612753 |
| 17 | (chronic disease* or noncommunicable disease* or non communicable disease*).mp. | 227877 |
| 18 | ((type II or type 2) adj diabetes).mp. | 166767 |
| 19 | (T2D or NIDDM or noninsulin or non insulin).mp. | 215417 |
| 20 | (myocardial infarction or myocardial isch?emia or stroke* or cerebrovascular infarction or cerebrovascular accident or musculoskeletal disease* or brain infarction).mp. | 732171 |
| 21 | cerebrovascular accident/ or coronary artery disease/ or breast cancer/ or breast tumor/ or colon tumor/ or colon cancer/ or musculoskeletal disease/ | 824966 |
| 22 | ((breast or colon or colorectal) adj3 (cancer* or tumo?r* or neoplasm*)).mp. | 718596 |
| 23 | ((coronary or heart) adj2 disease*).mp. | 579900 |
| 24 | Vascular Diseases/ or Peripheral Vascular Diseases/ | 39962 |
| 25 | peripheral occlusive artery disease/ | 34107 |
| 26 | neck pain/ or low back pain/ or backache/ | 110331 |
| 27 | OSTEOARTHRITIS/ | 81646 |
| 28 | postmenopause osteoporosis/ or osteoporosis/ | 116595 |
| 29 | (vascular disease* or peripheral vascular disease* or peripheral arter* disease*).mp. | 133543 |
| 30 | (low back pain or neck pain or back pain or osteoarthritis or osteoporosis).mp. | 347369 |
| 31 | 16 or 17 or 18 or 19 or 20 or 21 or 22 or 23 or 24 or 25 or 26 or 27 or 28 or 29 or 30 | 2749801 |
| 32 | body mass/ or body weight/ or weight control/ or weight gain/ or weight reduction/ or obesity/ or abdominal obesity/ or metabolic syndrome x/ | 976845 |
| 33 | (body weight or overweight or obes* or adipos* or body mass index or BMI or waist-hip ratio or waist circumference* or waist-height ratio).mp. | 1113520 |
| 34 | ((abdominal or body) adj2 fat).mp. | 68372 |
| 35 | blood pressure/ or diastolic blood pressure/ or systolic blood pressure/ or glucose blood level/ or glucose level/ or lipid/ or triacylglycerol/ | 862904 |
| 36 | cholesterol level/ or very low density lipoprotein cholesterol/ or cholesterol/ or high density lipoprotein cholesterol/ or cholesterol blood level/ or low density lipoprotein cholesterol/ or total cholesterol level/ | 313526 |
| 37 | adipose tissue/ or waist circumference/ or body fat/ or hypertension/ or glucose tolerance test/ or glucose tolerance/ or insulin resistance/ | 786392 |
| 38 | (blood adj (glucose or sugar* or pressure)).mp. | 693589 |
| 39 | (lipid* or triglycer* or hypertensi* or hyperglycemi* or glucose intoleran* or hyperinsulin* or insulin resistan* or cholesterol* or glyc?emic index or hypercholesterol* or metabolic syndrome).mp. | 1977024 |
| 40 | 32 or 33 or 34 or 35 or 36 or 37 or 38 or 39 | 3250278 |
| 41 | 31 or 40 | 5343782 |
| 42 | 3 and 15 and 41 | 11037 |
| 43 | limit 42 to (human and english language and exclude medline journals and embase and yr="2000 -Current" and (article or article in press) and journal and (adult <18 to 64 years> or aged <65+ years>)) | 1095 |

Note: # 3 indicates records related to South Asia

#15 indicates records related to physical activity

# 41 indicates records related to chronic disease or their markers

# 42 indicates records related to South Asia and physical activity and chronic disease/markers

Title of the database searched: PsycINFO 1806 to March Week 2 2018

Name of the software platform: Ovid

Date search was run: 18 March 2018

Limits used: English language, human, age group: 40+, journal articles

Search Strategy:

| # | Searches | Results |
| --- | --- | --- |
| 1 | (bangladesh* or bhutan* or india* or afghan* or nepal* or pakistan* or sri lanka* or srilanka* or maldiv* or south asia* or southasia*).mp. | 48480 |
| 2 | (physical activit* or physical inactiv* or exercise* or nonexerciser* or non exerciser* or muscle stretching exercise* or resistance train* or run* or jog* or swim* or walk* or stair* climb* or leisure activit* or recreation or danc* or gardening or game* or recreation* or sport* or physical fitness* or cycle* or bicycl*).mp. | 284348 |
| 3 | exercise/ or physical activity/ or aerobic exercise/ or yoga/ or physical fitness/ or running/ or motor performance/ or walking/ or sports/ or swimming/ or recreation/ or leisure time/ or screen time/ or daily activities/ or hobbies/ or holidays/ or active living/ or activity level/ or dance/ or television viewing/ or traveling/ or vacationing/ or computer games/ or lifestyle/ or active living/ or lifestyle changes/ or "activities of daily living"/ | 114105 |
| 4 | sedentary behavior/ | 1052 |
| 5 | (lifestyle* or life style* or sitting time or motor activity).mp. | 37832 |
| 6 | (sedentary adj1 (lifestyle* or activit* or behavio?r* or time)).mp. | 3113 |
| 7 | (computer* or smartphone* or mobile phone* or cell phone* or television* or TV).mp. | 149295 |
| 8 | (leisure adj3 activit*).mp. | 5829 |
| 9 | ((household or domestic) adj activit*).mp. | 399 |
| 10 | (leisure adj2 exercise*).mp. | 1065 |
| 11 | ((TV or television) adj3 (watch* or view*or time)).mp. | 2331 |
| 12 | ((screen or screen based) adj2 (time or view*)).mp. | 901 |
| 13 | television/ or mass media/ or mobile devices/ or cellular phones/ or computers/ or electronic communication/ | 33940 |
| 14 | 2 or 3 or 4 or 5 or 6 or 7 or 8 or 9 or 10 or 11 or 12 or 13 | 475590 |
| 15 | TYPE 2 DIABETES/ | 2988 |
| 16 | (chronic disease* or noncommunicable disease* or non communicable disease*).mp. | 10554 |
| 17 | ((type II or type 2) adj diabetes).mp. | 6605 |
| 18 | (T2D or NIDDM or noninsulin or non insulin).mp. | 643 |
| 19 | cerebral ischemia/ or Cerebrovascular Accidents/ or heart disorders/ or myocardial infarctions/ | 32431 |
| 20 | ((breast or colon or colorectal) adj3 (cancer* or tumo?r* or neoplasm*)).mp. | 14685 |
| 21 | ((coronary or heart) adj2 disease*).mp. | 11581 |
| 22 | (myocardial infarction* or myocardial isch?emia or stroke* or cerebrovascular infarction* or cerebrovascular accident or musculoskeletal disease* or brain infarction*).mp. | 36433 |
| 23 | (body weight or overweight or obes* or adipos* or body mass index or BMI or waist-hip ratio or waist circumference* or waist-height ratio).mp. | 66933 |
| 24 | back pain/ | 3544 |
| 25 | (vascular disease* or peripheral vascular disease* or peripheral arter* disease*).mp. | 1829 |
| 26 | (low back pain or neck pain or back pain or osteoarthritis or osteoporosis).mp. | 9458 |
| 27 | 15 or 16 or 17 or 18 or 19 or 20 or 21 or 22 or 23 or 24 or 25 or 26 | 152142 |
| 28 | body weight/ or overweight/ or underweight/ or weight gain/ or weight loss/ or body fat/ or body mass index/ or "obesity (attitudes toward)"/ or weight control/ | 26513 |
| 29 | obesity/ or body mass index/ or body fat/ or adipocytes/ or lipids/ or fatty acids/ | 30744 |
| 30 | (body weight or overweight or obes* or adipos* or body mass index or BMI or waist-hip ratio or waist circumference* or waist-height ratio).mp. | 66933 |
| 31 | ((abdominal or body) adj2 fat).mp. | 3838 |
| 32 | (lipid* or triglycer* or hypertensi* or hyperglycemi* or glucose intoleran* or hyperinsulin* or insulin resistan* or cholesterol* or fatty acid* or fatty tissue* or glyc?emic index or hypercholesterol* or metabolic syndrome).mp. | 39257 |
| 33 | hyperglycemia/ or blood pressure/ or diastolic pressure/ or systolic pressure/ or blood sugar/ or glucose/ or metabolic syndrome/ or hypertension/ or cholesterol/ or steroids/ | 23544 |
| 34 | (pressure adj (diastolic or systolic)).mp. | 199 |
| 35 | 28 or 29 or 30 or 31 or 32 or 33 or 34 | 110233 |
| 36 | 27 or 35 | 188357 |
| 37 | 1 and 14 and 36 | 549 |
| 38 | limit 37 to (human and english language and (360 middle age or "380 aged ") and "0110 peer-reviewed journal" and journal article and yr="2000 -Current") | 173 |

Note: # 1 indicates records related to South Asia

#14 indicates records related to physical activity

# 36 indicates records related to chronic disease or their markers

# 37 indicates records related to South Asia and physical activity and chronic disease/markers

Title of the database searched: EBM Reviews - Cochrane Central Register of Controlled Trials February 2018 
Name of the software platform: Ovid

Date search was run: 18 March 2018

Years covered: 2000 to Current

Limits used: English language, journal articles

Search Strategy:

| # | Searches | Results |
| --- | --- | --- |
| 1 | bangladesh/ or bhutan/ or india/ or nepal/ or pakistan/ or sri lanka/ | 2866 |
| 2 | (bangladesh* or bhutan* or india* or afghan* or nepal* or pakistan* or sri lanka* or srilanka* or maldiv* or south asia* or southasia*).mp. | 10336 |
| 3 | 1 or 2 | 10336 |
| 4 | exercise/ or muscle stretching exercises/ or resistance training/ or running/ or jogging/ or swimming/ or walking/ or stair climbing/ or leisure activities/ or recreation/ or dancing/ or gardening/ or games, recreational/ or video games/ or sports/ or physical fitness/ or bicycling/ | 22039 |
| 5 | (physical activit* or physical inactiv* or exercise* or nonexerciser* or non exerciser* or muscle stretching exercise* or resistance train* or run* or jog* or swim* or walk* or stair* climb* or leisure activit* or recreation or danc* or gardening or games, recreational or sport* or physical fitness* or cycle* or bicycl*).mp. | 116024 |
| 6 | "Physical Education and Training"/ or Motor Activity/ or life style/ or sedentary lifestyle/ | 8062 |
| 7 | (physical education or fitness* or motor activity).mp. | 11993 |
| 8 | (lifestyle* or life style* or sitting time).mp. | 11022 |
| 9 | (sedentary adj1 (lifestyle* or activit* or behavio?r* or time)).mp. | 1971 |
| 10 | cellular phone/ or television/ or Computers, Handheld/ | 512 |
| 11 | (computer* or smartphone* or mobile phone* or cell phone* or television* or TV).mp. | 34651 |
| 12 | (leisure adj3 activit*).mp. | 698 |
| 13 | ((household or domestic) adj activit*).mp. | 50 |
| 14 | (leisure adj2 exercise*).mp. | 82 |
| 15 | ((TV or television) adj3 (watch* or view*or time)).mp. | 210 |
| 16 | ((screen or screen based) adj2 (time or view*)).mp. | 279 |
| 17 | 4 or 5 or 6 or 7 or 8 or 9 or 10 or 11 or 12 or 13 or 14 or 15 or 16 | 153084 |
| 18 | coronary disease/ or coronary artery disease/ or myocardial infarction/ or Diabetes Mellitus, Type 2/ or chronic disease/ | 38097 |
| 19 | (chronic disease* or noncommunicable disease* or non communicable disease*).mp. | 15364 |
| 20 | ((type II or type 2) adj diabetes).mp. | 16956 |
| 21 | (T2D or NIDDM or noninsulin or non insulin).mp. | 11391 |
| 22 | Musculoskeletal Diseases/ or Breast Neoplasms/ or Colonic Neoplasms/ or stroke/ or brain infarction/ | 15338 |
| 23 | ((breast or colon or colorectal) adj3 (cancer* or tumo?r* or neoplasm*)).mp. | 32581 |
| 24 | (myocardial infarction* or myocardial isch?emia* or stroke* or cerebrovascular infarction* or cerebrovascular accident* or musculoskeletal disease* or brain infarction*).mp. | 57170 |
| 25 | ((coronary or heart) adj2 disease*).mp. | 26602 |
| 26 | (pressure adj (diastolic or systolic)).mp. | 1312 |
| 27 | vascular diseases/ or peripheral vascular diseases/ or peripheral arterial disease/ | 1227 |
| 28 | Low Back Pain/ or Back Pain/ or Neck Pain/ | 3667 |
| 29 | Osteoarthritis/ | 1506 |
| 30 | Osteoporosis, Postmenopausal/ or Osteoporosis/ | 3051 |
| 31 | (vascular disease* or peripheral vascular disease* or peripheral arter* disease*).mp. | 4710 |
| 32 | (low back pain or neck pain or back pain or osteoarthritis or osteoporosis).mp. | 24402 |
| 33 | 18 or 19 or 20 or 21 or 22 or 23 or 24 or 25 or 26 or 27 or 28 or 29 or 30 or 31 or 32 | 166314 |
| 34 | body weight/ or overweight/ or underweight/ or weight gain/ or weight loss/ or body fat/ or body mass index/ or "obesity (attitudes toward)"/ or weight control/ | 20377 |
| 35 | obesity/ or body mass index/ or body fat/ or adipocytes/ or lipids/ or fatty acids/ | 21416 |
| 36 | (body weight or overweight or obes* or adipos* or body mass index or BMI or waist-hip ratio or waist circumference* or waist-height ratio).mp. | 65093 |
| 37 | ((abdominal or body) adj2 fat).mp. | 5461 |
| 38 | (lipid* or triglycer* or hypertensi* or hyperglycemi* or glucose intoleran* or hyperinsulin* or insulin resistan* or cholesterol* or fatty acid* or fatty tissue* or glyc?emic index or hypercholesterol* or metabolic syndrome).mp. | 100023 |
| 39 | hyperglycemia/ or blood pressure/ or diastolic pressure/ or systolic pressure/ or blood sugar/ or glucose/ or metabolic syndrome/ or hypertension/ or cholesterol/ or steroids/ | 51676 |
| 40 | (pressure adj (diastolic or systolic)).mp. | 1312 |
| 41 | 34 or 35 or 36 or 37 or 38 or 39 or 40 | 163439 |
| 42 | 33 or 41 | 289516 |
| 43 | 3 and 17 and 42 | 658 |
| 44 | limit 43 to (journal article and yr="2000 -Current" and english language) | 421 |

Note: # 3 indicates records related to South Asia

#17 indicates records related to physical activity

# 42 indicates records related to chronic disease or their markers

# 43 indicates records related to South Asia and physical activity and chronic disease/markers
