# Supplementary material for: Physical activity participation and the risk of chronic diseases among South Asian adults: protocol for a systematic review and meta-analysis
Source: Syst Rev. 2018 Oct 30;7:177. doi: 10.1186/s13643-018-0848-9 (PMC6208083; doi:10.1186/s13643-018-0848-9)
Supplement: Supplementary file 3 — Microsoft Excel data extraction template. (DOCX 13 kb) [file 13643_2018_848_MOESM3_ESM.docx]

**Data extraction template**

| Study ID |  |
| --- | --- |
| Title |  |
| Author |  |
| Year of publication |  |
| Country |  |
| Study population | Age, gender |
| Study design |  |
| Sample size |  |
| Sample size calculation |  |
| Study power |  |
| Sampling technique |  |
| Response rate |  |
| Data source | Survey, secondary analysis |
| Exclusion criteria |  |
| Outcome variables |  |
| Diagnosis |  |
| Disease classification |  |
| Physical activity domain studied |  |
| Assessment technique |  |
| Recall period |  |
| Reporting measure | METs or minutes/week or Yes/No |
| Physical activity categories |  |
| Validity and reliability |  |
| Measure of association |  |
| Disease prevalence |  |
| Association estimates | 95% CI, p-value |
| Direction of association |  |
| Adjusted/unadjusted estimates |  |
| Key confounding variables adjusted |  |
| Strengths of the study |  |
| Limitations of the study |  |
| Conclusion |  |
| For case-control studies |  |
| Selection of cases |  |
| Selection of controls |  |
| For cohort studies |  |
| Follow up period |  |
| Loss to follow up |  |
